# Supplementary material for: New dual method for elastica regularization
Source: PLoS One. 2022 Mar 15;17(3):e0261195. doi: 10.1371/journal.pone.0261195 (PMC8923477; doi:10.1371/journal.pone.0261195)
Supplement: Attachment — Submitted filename: Response to Reviewers.pdf [file pone.0261195.s001.pdf]

Original Manuscript ID: PONE-D-21-19897

Original Article Title: **“**New Dual Method for Elastica Regularization”

Dear Editor,

Many thanks to the editor and the reviewer for affirming our work and pointing out the potential issues behind the Lena image. We apologize for our misuse of the image and have removed it from our paper to comply with the standards of the PLOS ONE journal. We have also carefully revised our manuscript according to the comments.

Thank you again for your feedback and consideration.

Best Regards,

<Jintao song> et al.

Concern # 1: We note that in this manuscript you have presented an image known as Lena/Lenna,which has a problematic history, please see https://en.wikipedia.org/wiki/Lenna for more information. We do not feel that this image is in line with the values of PLOS ONE, and would therefore request that you at this point substitute this image in the manuscript with another image.

Author response: We have removed the experiments in the image denoising and inpainting sections using the Lena image and replaced them with new experiments using the Baboon and the Cameraman image, respectively.

Author action: In Fig.6, Fig.7, and Table 1, we replaced the Lena experiment with experiments on the Baboon image. In Fig.8, we replaced the Lena experiment with an experiment on the Cameraman image.

Concern # 2: Overall, the paper appears to be well written, but there were a few minor typos throughout, such as "relevent error" which should probably be "relative error." Please be sure to do a final careful proof-reading of for the final version of the manuscript.

Author response: Thanks a lot for this suggestion, we have changed “relevant error” into “relative error”. We have also carefully proofread the final manuscript to ensure that similar problems do not occur.

Author action: In Fig.1 and Fig.6, we changed “relevant error” into “relative error”.
